# Improving the Nutrient Quality of Foods and Beverages Using Product Specific Standards for Nutrients to Limit Will Substantially Reduce Mean Population Intakes of Energy, Sodium, Saturated Fat and Sugars towards WHO Guidelines

**DOI:** 10.3390/nu14204289

**Published:** 2022-10-14

**Authors:** Mariska Dötsch-Klerk, Eva M. R. Kovacs, Ujwal Hegde, Ans Eilander, Julie I. Willems

**Affiliations:** 1 Unilever Foods Innovation Centre, 6708 WH Wageningen, The Netherlands; 2 Unilever R&D, Colworth Science Park, Sharnbrook MK44 1LQ, UK

**Keywords:** nutrient profiling, food quality, nutritional quality, reformulation, modelling, sugar reduction, salt reduction

## Abstract

Background: International strategies to reduce chronic diseases have called for a reduction in the amounts of saturated fat (SAFA), trans fat (TFA), salt and sugars in the global food supply. This paper describes the development approach and potential impact of a set of standards for these nutrients to drive food (re)formulation. Methods: To set the standards, WHO nutrient guidelines for daily intake were translated into product group specific standards. The impact of reformulation towards these standards on population nutrient intakes was modelled using the food consumption data of five countries: UK, France, US, Brazil and China. The impact of the TFA standards could not be modelled due to lack of data. Results: (Re)formulation of foods and beverages towards these standards would substantially decrease mean population intakes of energy, sodium, SAFA and sugars, with reductions up to 30%. Conclusions: These science-based standards for nutrients to limit could drive impactful reductions in energy, sodium, SAFA and sugars in food and beverage products, enabling mean population intakes to move closer to WHO nutrient guidelines.

## 1. Introduction

Worldwide there is a high prevalence of chronic diseases related to overconsumption of energy dense diets with poor nutrient quality. Studies have clearly shown that diets high in calories, sodium, SAFA or sugars are associated with increased risk of chronic diseases, mortality and disability-adjusted life-years (DALY’s) [1,2]. WHO launched their Global Strategy on Diet, Physical Activity and Health in 2003 [3], followed by their Global Action Plan for the Prevention and Control of Noncommunicable Diseases 2013–2020 [4], including a call for industry to reduce the amounts of saturated fat (SAFA), trans fat (TFA), salt and sugars in the global food supply.

In response, Unilever implemented its own nutrient-profiling approach to evaluate and improve the nutritional quality of the Unilever foods and beverages portfolio in 2003, focussing on these above-mentioned nutrients to limit, and approach for setting product nutrient standards was published [5]. Since the launch, these standards have guided the company’s (re)formulation agenda and related commitments. The standards are regularly updated to stay aligned with scientific developments and to ensure further product reformulation. For example, in 2009 the sodium standards were revised and the potential impact was modelled using dietary survey data [6]. The modelling showed that application of the standards would substantially reduce population sodium intakes, enabling intakes to be in line with the WHO recommended 5 g salt (2000 mg sodium) per day [7].

Nowadays, there are numerous nutrient profiles schemes used around the globe and for different purposes, such as reformulation, marketing restrictions and front-of-pack labelling. However, these schemes are often focused on one nutrient or are based on limited product groups not covering all foods eaten by consumers. In addition, the use of generic criteria across all products per 100 g or 100 mL in some of these schemes would not encourage reformulation of the core of the company’s product portfolio. For a global food company, it is important to set stretching, yet feasible, product group specific standards to encourage foods and beverages worldwide to be (re)formulated to maximize the public health impact.

The Unilever standards for nutrients to limit continue to form the solid basis for (re)formulation, as well as responsible marketing principles, nutrition and health claims eligibility, and recipe development. The scope of the current paper is to describe the principles and development approach of the revised standards. Moreover, the potential impact of reformulation towards these standards on nutrient intakes has been evaluated through modelling on food consumption data of five different countries, representing different regions across the globe: United Kingdom, France, United States, Brazil and China.

## 2. Materials and Methods

### 2.1. Standards Development Approach

**Figure 1:**
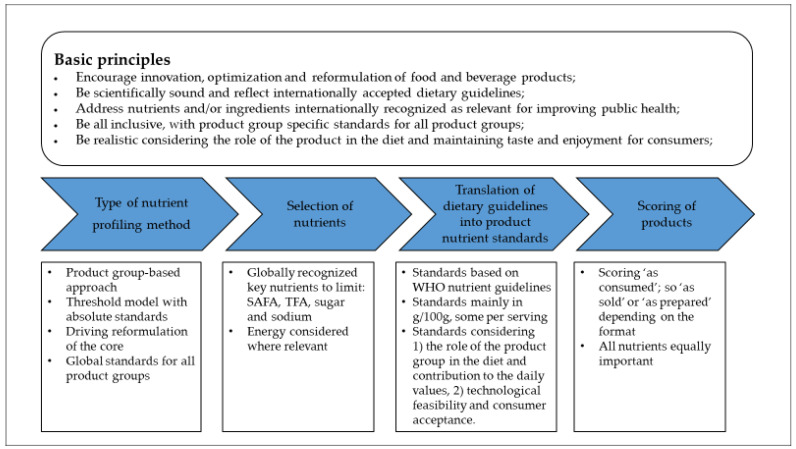
Schematic approach for setting the Unilever standards for nutrients to limit.

The approach used for setting the product nutrient standards is schematically shown in Figure 1 and further explained below.

#### 2.1.1. Basic Principles

To ensure nutrient profiles are an effective and credible instrument to help create better diets, five global principles were defined to which our nutrient profiling scheme should adhere (Figure 1). These principles were taken into account when setting the standards.

#### 2.1.2. Type of Nutrient Profiling Method

Based on long-standing expertise in reformulating products, we moved over time from a mainly generic nutrient profiling system, as introduced in 2003 [5], to a product group specific nutrient profiling scheme that considers the role of the product in the diet. The product groups were defined in alignment with the company’s current product portfolio. The standards follow a threshold model with absolute standards (thresholds) rather than relative reduction targets. To achieve the biggest impact on public health, the standards are set at a level driving reformulation in the core of the portfolio, rather than focusing on niche products.

#### 2.1.3. Selection of Nutrients

In line with international strategies of the WHO [3,4] and the United Nations declaration [8], SAFA, TFA, sodium, and sugars were selected as key nutrients to limit. In addition, we defined energy standards for prominent calorie delivering products. The starting point for the setting of the standards were the WHO guidelines for energy, SAFA [9], sodium [7] and sugar intake [10] (Table 1). Absolute daily guidelines used for SAFA and sugars were based on a 2000 kcal diet, in line with regulation for daily value labelling on-pack [11].

#### 2.1.4. Translation of Dietary Guidelines into Product Nutrient Standards

The product group specific standards were set considering the role of the product group in the diet, including appropriate serving size and frequency of consumption, and their contribution to the WHO dietary guidelines. In addition, technological feasibility, but also taste and consumer acceptance were taken into account. To encourage consumer acceptance the most preferred solution to reduce sodium and sugars in foods is gradual reduction. However, this takes time and is difficult to apply in some products groups because of structural properties, such as salt in bread or sugars in ice-cream. Other solutions available to reduce sodium or sugar in foods are the use of salt substitutes or sweeteners. However, these can also only be used to certain extent as they do not have the same physical properties as salt or sugars. Another limitation for using “artificial” salt replacers or sweeteners is the increasing consumer interest in naturalness with a demand for short ingredient lists with recognizable ingredients. In addition, some nutrient-profiling schemes are actively discriminating against the use of additives.

Most standards are expressed in g/100 g or g/100 mL. This is the most common way of expressing nutritional composition in food composition tables and on-pack labelling worldwide, and therefore generally easy to interpret. However, the reference unit for energy was set per serving to ensure that the absolute energy value of a product would need to be below a specific cap. An exception was Ice-cream and Desserts and Snacks—Sweet and Savoury, where all standards were set per serving. This is because providing portion-controlled choices is a key strategy to offer products with less sugars and fewer calories, especially for pre-packed, single-serve products such as an ice cream stick. Generic information about how product group specific standards per nutrient were set is provided below.

##### Energy

The energy standards are based on the daily energy guidelines for women (2000 kcal/day), but also men (2500 kcal/day) and children aged 6–12 years (averaged to 1855 kcal/day). Maximally 10% of energy is recommended to come from beverages [12]. The remaining 90% of energy was allocated as follows: breakfast (20%), lunch (30%), dinner (30%), and three snacks (20%) (Supplementary Table S1). Energy standards were defined for prominent calorie delivering product groups, defined as generally delivering 15% of the recommended daily value (DV) or more, such as meals (Main Meals; Small Meals), dishes (Carbohydrate-based Dishes, Animal Protein, Plant Protein) and snacks (Ice-cream and Desserts; Snacks—Sweet and Savoury). For beverage product groups, no energy standards were set as this is largely covered by the sugar standard.

##### Sodium

Already in 2009, sodium standards were set to target the WHO recommended maximum intake of 5 g/day of salt (2000 mg/day of sodium). Data modelling using food consumption survey data from the United States, United Kingdom and The Netherlands showed that cross-industry food product reformulation towards these standards would indeed enable reducing sodium intakes towards the WHO guideline of 2000 mg/day [6]. Therefore, these sodium standards were continued. However, a few newly introduced product groups (e.g., Plant-based Protein) were not part of that modelling exercise, hence for these new product groups, standards were set considering the daily value contribution based on a reference serving.

##### SAFA

Standards are based on the WHO recommendation to limit intake of SAFA to maximally 10% of the total energy consumed, which equals 20 g per day. Standards that used to be expressed as percentage of energy in the past were converted to g/100 g, which better equip product developers to assess products during (re)formulation design. Exceptions are the SAFA standards for Emulsion-based Sauces and Cooking Fats, and Mustard, where energy is mainly delivered by fat. For these product groups, fat quality is considered more relevant than SAFA quantity as such, hence standards were set as percentage of total fat, considering the SAFA levels of healthy oils which is around 18%.

##### Sugars

In contrast to the previous standards for nutrients to limit, sugars standards were now set for all product groups. The WHO recommends limiting free sugar intake to ≤10% of total energy intake, which equates to approximately 50 g of free sugars per day. Science supports the differentiation and greater relevance of free versus total or added sugars from a public health perspective. However, the main challenge to implement free sugars in standard setting is the lack of an agreed technical specification of free sugars. Therefore, sugar standards were set either as total or added sugars, in line with food composition tables and nutrition labels that also feature total and/or added sugars. For most of the product groups the standard was set for added sugars, not to limit the inclusion of plant-based ingredients while limiting the use of added sugars for sweetening or other technical purposes. For Main Meals and Small Meals, the standard was set at a level allowing maximally 10% of the daily value for free sugars per serving. For most of the meal components the standard would allow a maximum of 5% of the daily value per serving. For product groups such as Ice cream and Desserts, Snacks—Sweet and Savoury, RTD and Concentrated Beverages, and Cereal and Malt-based Beverages, the standard was set for total sugars to avoid use of high sugar containing ingredients (such as fruit concentrates) that are not counted as added sugars. These standards were set a level allowing maximally 25% of the daily value for free sugars per serving.

##### Trans Fat

In addition to the product group specific standards for energy, sodium, SAFA and sugars, all products need to comply with our standards for TFA: Partly hydrogenated vegetable oil (PHVO ≤ 1 g/100 g (as sold) and industrial TFA (iTFA) ≤ 2 g/100 g fat (as sold) [13].

#### 2.1.5. Scoring of Products

Products should preferably be scored ‘as consumed’ as this is most realistic and relevant for the consumer. This means products are scored ‘as sold’ if they do not need the addition of other ingredients to enable consumption, or if they can be used in different amounts and in a variety of dishes. Products are scored ‘as prepared’ if they are sold in a dry or concentrated form and need reconstitution, or if fresh ingredients need to be added. If no preparation data is available, the product is by default scored as sold. TFA standards are always applied to products as sold.

An overview of the product groups and standards is provided in Supplementary Table S2.

### 2.2. Modelling

To evaluate the potential impact of our standards on population nutrient intakes, modelling was conducted using complete food consumption survey data. Analyses were performed by Exponent International Ltd.(Harrogate, UK) between December 2021 and March 2022.

#### 2.2.1. Food Consumption and Composition Data

Five countries were included in this analysis based on the availability of recent nutrient intake data and representation of different regions: Europe (UK—NDNS and France—INCA3) [14], South America (Brazil—INA) [15], North America (US—NHANES) [16] and Asia (China—CHNS) [17]. An overview of the food consumption surveys and food composition tables used in the assessments is presented in Table 2. In the surveys from the US, China and France, the ages of individuals ranged from birth to >79 years. The UK survey included individuals from 1.5 years and older, and the Brazilian survey 10 years and older. As the standards are not designed to include products for infants and toddlers, nutrient intake data for individuals aged ≥2 years only were included in the model for the UK, France, the US and China, whereas for Brazil data of all individuals were included.

The US survey had data on all nutrients available. For the Chinese survey only data on energy and sodium intake were available, so analysis was restricted to these two nutrients. For the UK survey, data for non-milk extrinsic sugars (NMES) was used as proxy for added sugars [18]. The Brazilian survey did not include total sugar, so all sugar standards, including the total sugar standards defined for the different snacks and beverage categories, were applied to added sugar values. The French survey did not include data on added sugars and therefore all sugar standards, including those defined as added sugar standards, were applied to total sugar levels. The impact of the TFA standards could not be modelled as PHVO and iTFA data are not present in food composition tables. Moreover, levels in food products are in general already very low due to successful reformulation efforts over the last decades.

#### 2.2.2. Food Group Alignment

Before scenario calculations could be performed, food groups in the food composition tables of the selected countries needed to be aligned with the Unilever product groups for which the different product specific standards were defined (Supplementary Table S2). The Unilever standards do not include standards for fat spreads and cheese products as these are no longer relevant to the portfolio. However, as these food groups can provide a substantial contribution to SAFA and/or sodium intake in some countries, previously used standards were applied (Supplementary Table S3). There are also no specific standards for dairy, fruits and vegetables, so these were left unchanged. Lastly, nutrient values of alcoholic beverages, sugars/sweeteners, salt and nutritional supplements were not altered.

Ingredients from recipes/dishes were not separated. For example, if a respondent reported consumption of ‘Spaghetti Bolognese’, the food was classified under the food group Main Meal. However, if a respondent reported consumption of spaghetti sauce and pasta as separate items, the items were categorised as Meal Sauces and Carbohydrate-based Dishes, respectively. All database mappings were checked by several people from Exponent and Unilever to ensure alignment and consistency across databases.

#### 2.2.3. Modelling Scenario’s

Following the food group alignment, the next step was to review the food composition data regarding the target nutrients to set up the nutrient models for each survey database. For each of the countries two scenarios were analysed:

- Baseline: Population nutrient intake based on the original survey data
- Reformulation: Population nutrient intake following ‘hypothetical reformulation’ to meet the product group specific nutrient standards defined

To calculate the current or ‘baseline’ population nutrient intake in the surveys, a standard assessment was run with the survey data without changes in nutrient levels in the original food composition data. To model the potential impact of reformulation, nutrient values in the food composition tables for the products that were not compliant to the standards were modified to simulate reformulation, assuming all food products consumed in each survey would be compliant with the relevant product group standards. This means that the nutrient values for energy, saturated fat, sodium, total and added sugars in the food composition tables for the target food products were replaced (as necessary) by the value of the relevant standard, which resulted in a reduced value.

For the surveys where both total and added sugars values were available, changes were mutually adjusted to avoid that added sugars values could be higher than total sugars values in a product. In addition, a reduction in added sugars was assumed to not be compensated for by the use of sugar containing ingredients not counted as added sugars (e.g., dried fruit or fruit concentrates), in line with Unilever’s reformulation guidance.

#### 2.2.4. Conversion Standards in g/serving to g/100 g

Most of the standards express the nutrients for each food product as grams per 100 g, which is consistent with the food composition data as used in the different survey databases. However, there were some exceptions to this:

- The standard value for Energy is expressed per serving of each food product.
- The standard for Saturated Fat in the food groups, Emulsion-based Sauces, and Mustards, is expressed as % of total fat.
- The nutrient standards for the food groups Ice cream and Desserts, and Sweet and Savoury Snacks, are per serving.

As such, serving size for foods aligned with those categories were defined as required, using reference serving sizes suggested by Unilever, where available (e.g., 300 g for Small Meals). For foods with more varying serving sizes (i.e., Snacks—Sweet and Savoury, and Ice-Cream and Desserts), serving sizes were obtained on a country specific basis [19,20,21,22,23,24,25]. Using these serving sizes, standards expressed per serving were converted to a standard per 100 g, which were subsequently used in the reformulation scenario.

#### 2.2.5. Data Analysis

The estimated mean nutrient intakes for the baseline and reformulation scenarios were conducted using dietary exposure software relevant for each survey database. The estimated mean nutrient intake for the baseline and reformulation scenarios for UK, Brazil, and France were conducted in DaDiet Software of Dazult, Maynooth, Ireland [26]. This is a web-based software tool that allows accurate estimation of exposure to nutrients and to substances added to foods, including contaminants, food additives and pesticides. For the US and China, different software packages were used, as these were performed by local teams of Exponent that do not work with DaDiet. The US estimates were derived using Exponent’s Foods Analysis and Residue Evaluation Program (FARE^®^ version 14.06, Washington DC, US) software. For China, the estimated mean nutrient intakes for the baseline and reformulation scenarios were conducted using STATA (version 12.1, 2011, StataCorp LP, College Station, TX, USA). The primary outcome for the analysis was the percentage change per target nutrient in the reformulation scenario compared to the baseline scenario. Population mean nutrient intakes were calculated using ratio estimation and nonparametric techniques, incorporating survey weights where appropriate to provide representative intakes for specific population groups. Outcome measures were calculated for the total diet and per food group. Mean population intakes at baseline and after hypothetical reformulation were plotted against the WHO nutrient guidelines shown in Table 1. As WHO does not provide a guideline for total sugars, we used 90 g/day in line with EU regulation for daily value labelling on-pack [11].

## 3. Results

**Figure 2:**
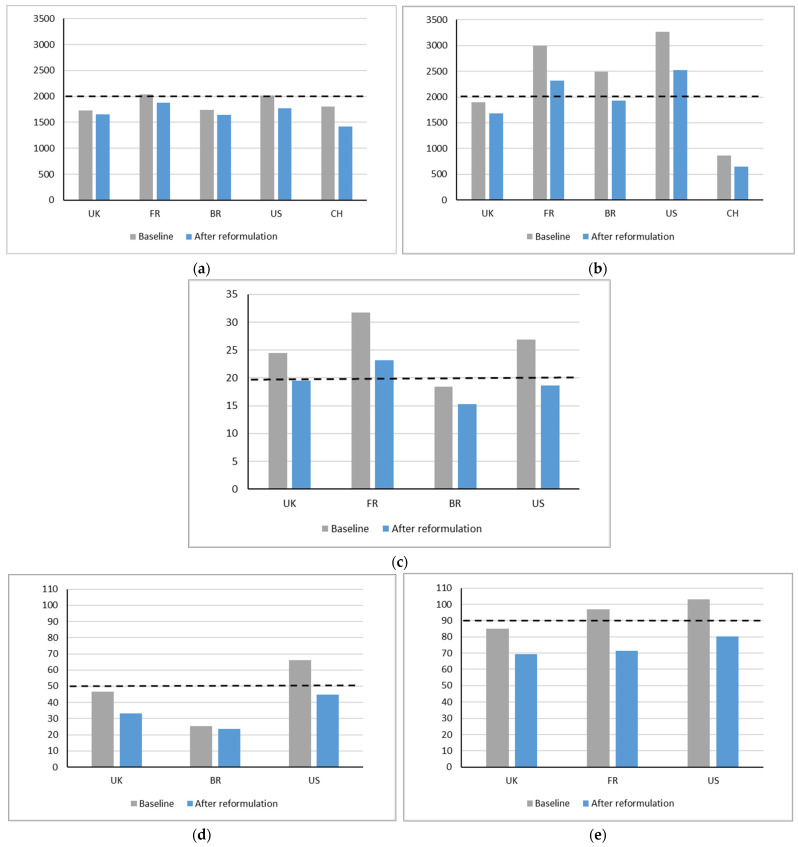
Mean population intakes at baseline and after hypothetical reformulation for (**a**) Energy (kcal), (**b**) Sodium (mg), (**c**) SAFA (g), (**d**) Added sugars (g), and (**e**) Total sugars (g). The grey line represents the WHO nutrient guideline (Table 1). For total sugars, 90 g/day was used as guideline in line with EU regulation for daily value labelling on-pack [11].

For all surveys included in the modelling, overall results show that reformulation of food products towards Unilever’s standards for the nutrients to limit would reduce mean population intakes of these nutrients (Figure 2a–e). In most cases, reformulation would bring mean population nutrient intakes close to or below the WHO nutrient guidelines for maximal intake. For France and the US, the WHO guidelines were not fully achieved for all nutrients; however, reductions were substantial.

### 3.1. Energy

For energy, projected reductions in intake would range from −4% for the UK to −21% for China (Figure 2a). It must be noted that for all surveys baseline energy intakes were already close to or below the recommended daily value. From the seven food groups with energy standards, the top five accounted for ca 90–100% of the reduction in intakes in all countries (Supplementary Table S4). In all countries, this top five included Small Meals, Carbohydrate-based Dishes, and Snacks—Sweet and Savoury, and in four countries Main Meals was in the top five. Hypothetical reformulation of Small Meals accounted for most of the reduction in intakes for UK, Brazil and US. For France it was the Snacks—Sweet and Savoury with the largest contribution, while for China it was the Carbohydrate-based Dishes.

### 3.2. Sodium

Reductions in sodium intake after hypothetical reformulation were similar for the French, Brazilian, the US and Chinese survey averaging 23–24% (Figure 2b). An exception was the UK survey where the projected reduction was lower at 11%, but in this survey mean population sodium intake was already below the WHO recommended value of 2000 mg/day at baseline. For China, sodium intake at baseline as measured in the survey was also below the WHO guideline, and despite the substantial relative reduction in sodium intake after hypothetical product reformulation, the absolute reduction was smaller when compared to the other countries. For Brazil, reformulation would help to bring mean population sodium intake below 2000 mg/day. For France and US, mean population intakes would still be higher than 2000 mg/day, despite the considerable relative reductions observed. The top five food groups contributing most to reductions in sodium intakes accounted for 67% in the UK survey to up to 95% for China (Supplementary Table S5). In all five countries Small Meals, Bread products and Animal Protein, in four countries Main Meals, and in three countries Carbohydrate-based Dishes were among the top five food groups contributing most to sodium intake reduction after hypothetical reformulation. Other food groups featuring in the top five were: Cheese Products for UK, Soups for France, and Pickled and Fermented Vegetables for China.

### 3.3. SAFA

For SAFA, the projected reductions in intake were also substantial, but lower for UK (20%) and Brazil (17%) than for France (27%) and US (30%) (Figure 2c). In all four countries, population mean intakes after hypothetical reformulation were close to or below the WHO daily guideline of ~20 g/day. The top five food groups contributing most to reductions in SAFA intakes accounted for 65% for France to nearly 100% for Brazil (Supplementary Table S6). In all four countries, Small Meals and Animal Protein, in three countries Main Meals and Snacks—Sweet and Savoury, and in two countries, Cheese Products and Fat Spreads were among the top five food groups contributing most to SAFA intake reduction after hypothetical reformulation. Other food groups featuring in the top five were Bread Products (Brazil) and Carbohydrate-based Dishes (US).

### 3.4. Added Sugars

For added sugars, intake data was available for only three of the surveys, UK, Brazil and US (Figure 2d). For the UK and the US, reductions in added sugars after hypothetical reformulation were both around 30%, and mean population intakes dropped below the WHO guideline of 50g/day. In the Brazilian survey, baseline mean population intake of added sugars was already below the recommended maximum value and decreased 7% after hypothetical product reformulation. The top five food groups contributing most to reductions in added sugars accounted for 81% of the total change for the UK and 85% for the US (Supplementary Table S7). Cereals, Snacks—Sweet and Savoury, and RTD and Concentrated Beverages, were among the top five in both the UK and the US. In the UK also Spreads—Sweet and Savoury, and Fruit and Vegetable Juices featured in the top. In the US, Small Meals, Animal Protein, but also Toppings—Sweet and Savoury, were among the top five. In Brazil, it was mainly Snacks—Sweet and Savoury, and Fruit and Vegetable Juices accounting for 94% of the reduction in added sugar intake.

### 3.5. Total Sugars

For total sugars, the impact of sugar reduction could only be modelled for UK, France and US (Figure 2e). In the UK and the US, hypothetical reformulation reduced intakes with about 20%, and mean population intakes dropped below the DV of 90 g used for labelling purposes. For the UK and the US, the contribution of the top five of food groups most contributing to the change showed the same picture as for added sugars (Supplementary Table S8). In France, the reduction was slightly larger with 26%. For France, the top five contributors accounting for most of the change in total sugars included Fruit and Vegetable Juices, Snacks—Sweet and Savoury, Spreads—Sweet and Savoury, RTD and Concentrated Beverages, and Ice-cream and Desserts.

## 4. Discussion

Unilever’s standards for nutrients to limit, that are used to drive reformulation, were recently revised based on nearly 20 years of experience in nutrient profiling. The potential impact of reformulation towards these updated standards on nutrient intakes was modelled using food consumption survey data of the United Kingdom, France, the United States, Brazil and China. For all surveys, modelling results showed that reformulation of food products towards these standards would substantially reduce mean population intakes of energy, sodium, saturated fat and sugars, and where this was not the case yet, bring them closer to the WHO recommended daily intake value. Overall, a rather small number of food groups accounted for a large proportion of reductions in energy, sodium, and saturated fat and sugar intake. Small Meals was a top source of potential reductions in energy, sodium, and saturated fat across all surveys, while Animal Protein was a top source for potential reductions in sodium and saturated fat. For added and total sugars, the results were more mixed across the different countries, but overall changes in Snacks—Sweet and Savoury, Fruit and Vegetable Juices, and RTD and Concentrated Beverages, seemed to drive potential reductions in sugar intake most.

Unilever’s nutrient profile includes standards for all relevant nutrients to limit, whereas government endorsed programmes for reformulation often address one specific nutrient (e.g., salt or sugars) and they tend to focus on the main contributors to intake in the country, such as salt in bread in European countries versus soy sauces in Asian countries. Experience has taught that it is important to set stretching, yet feasible, standards considering technological feasibility, but also consumer acceptance. This ensures the standards will encourage reformulation in the majority of products in a product group in order to maximize the public health impact. The standards and reformulation progress are audited and the approach to reformulation has been recognised by the Access to Nutrition Initiative’s Global Index [27] and more recently by the new World Benchmarking Alliance’s Food and Agriculture Benchmark [28]. Therefore, the standards and methodological approach can serve as good input in external stakeholder discussions on nutrient profiling and standard setting.

The modelling included most recent individual, nationally representative food consumption survey data on different population groups across different global regions. Although no survey data were available for countries in Africa, and for China the data was restricted to energy and sodium only, the results do give a good indication of the potential impact of food reformulation that could be achieved across the globe. However, we acknowledge that the modelling represents a theoretical approach and results are dependent on the quality of the input data and assumptions made.

Well-known limitations of consumption surveys are under-reporting or misreporting of food consumption, food coding errors, subject sampling bias, and other sources of uncertainty [29]. In addition, the time period in which survey data was obtained varied between the individual countries and may have impacted nutrient intakes as food consumption patterns evolve over time depending on trends, changing awareness of environmental and health impacts of different foods, affordability and availability of certain foods [30].

Dietary surveys generally do not accurately account for discretionary sodium added by consumers after preparation, and therefore total sodium intakes may be underestimated. The impact is biggest for China, where mean population sodium intake at baseline as measured in the survey was far below the WHO guideline of 2000 mg/day, and the absolute impact of hypothetical reformulation was only 208 mg, despite a relative reduction in intake of 24%. The values for sodium in the Chinese survey are not a good reflection of actual sodium intake in the Chinese population, as other studies have shown that in China most of the sodium comes from discretionary sources such as table salt and other condiments, which were not included in the survey [31]. A recent paper showed that adding salt to foods at the table is associated with a higher hazard of all-cause premature mortality and lower life expectancy [32]. The same counts for intake of added sugars in Brazil. In the Brazilian survey, baseline added sugar intake was already below the WHO recommend 50 g/day, and both relative and absolute impact of hypothetical reformulation was only small (6% = 2 g). This could partly be explained by the fact that baseline added sugar levels in foods and beverage products in the Brazilian food composition database used in the survey, were already lower than for similar products in the databases used in the UK and the US survey. However, more important is that in Brazil most of the sugar intake is coming from discretionary sources [15], which is not accurately included in the added sugar variable of the Brazilian food composition database.

As mentioned, the Brazilian survey only included a variable for added sugars, and therefore standards defined for total sugars were applied to added sugars instead. This may have led to an under-estimation of added sugar intake after reformulation (and over-estimate potential changes), but the impact is likely small as the sugars in snacks and ice-cream, and sugar-sweetened beverages, are predominantly added sugars. The French survey only had data available for total sugars and, therefore, all standards applied were to total sugars. This may have led to an under-estimation of total sugar intake after reformulation (and over-estimate potential changes) as this approach assumes that all sugars in foods is added sugars and does not account for intrinsic sugars such as those found in fruits and vegetables or dairy products.

Except for sugars, where changes in either total or added sugars were mutually adjusted for if both were available, the impact of the different standards was assessed independently. This implies that for food groups which had no separate standard for energy, reductions in SAFA or sugars were not translated to changes in energy content. This is a conservative scenario, assuming SAFA and sugar reduction does not necessarily lead to energy reduction as replacements can provide similar calories.

The UK, France, Brazil and the US surveys used a “foods-as-consumed” approach in which food codes correspond to mixed dishes in the dataset. This means that ingredients from recipes could not be separated and, therefore, the surveys included a relatively high number of meals and dishes. Dishes were often mixtures of different components (meat, carbohydrates, vegetables) and it was not clear whether there was one clear main component making up over 70% of the meal or dish or if it was a mixture of components in more equal amounts. Therefore, in these cases, decisions were made on classifications in alignment with the people involved in the modelling.

Some of the product nutrient standards were set on a per serving basis, while nutrient composition data are presented as per 100 g for each survey. For the modelling, these standards had to be converted to per 100 g using a serving size estimation. The serving sizes were estimated on a per country basis, as this was considered most representative and therefore varied greatly between countries. This may have resulted in differences between countries in the nutrients expressed per 100 g. In practice, it could mean that reformulation in some country might be more difficult than another country. However, alternative to reformulating the product, modification of the product size (i.e., reduction in size) could prove useful to adapt the product to meet pre-defined nutrient standards.

For France and the US, results of the reformulation scenarios showed that mean population sodium intakes would not yet reach the WHO guideline of maximally 2000 mg/day. Results confirm that a multi-stakeholder approach is needed to reduce intakes of all relevant nutrients to below the recommended maximum levels in every country. Next to product reformulation, also behaviour change strategies are needed to help consumers adapt their diets and reduce intake of food products high in SAFA, sodium or sugars, but also use of discretionary sources of sodium and sugars [33,34]. This is also clearly shown by the results from the UK survey. In this survey, mean population sodium intake at baseline was already below the WHO guideline of 2000 mg/day. These results reflect the success of the national salt campaign in the UK, which included both reformulation targets and a public health awareness campaign [35].

Nevertheless, the substantial relative reductions in intakes in all countries observed in our modelling study indicate that industry-wide reformulation of a country’s entire food supply, towards defined standards will help to reduce overall nutrient intakes. Moreover, these changes in nutrient intakes are likely to have an impact on public health outcomes. Various modelling studies have shown that food reformulation leading to reductions in intakes of energy, sodium, SAFA, and sugars could reduce risk of chronic diseases [36,37,38,39]. A recent review of modelling studies revealed that the evidence is stronger for sodium interventions, but less conclusive for sugars and fat reformulations [40].

Within Unilever, the application of standards for nutrients to limit has already driven substantial progress in terms of product improvement. In 2020, 61% of the product portfolio met all nutrient standards, achieving the commitment of 60% made in 2010 [41]. In total, 37 million tonnes of salt were removed from food products, over 15,000 tonnes of sugars from ice creams, and around 680,000 tonnes of sugars from ice-tea drinks, in that same period [42]. We have now extended the commitment to 70% by end of 2022 [43] and we are not focusing only on nutrients to limit. It is also important to increase intakes of nutrients and ingredients that have been associated with a positive impact on public health [1,2]. Therefore, we have also defined product standards for positive ingredients and nutrients. The ingredients and nutrients were selected in line with dietary guidelines and recommendations for a healthy and sustainable, and thus a more plant-based diet [44]. Positive ingredient standards include vegetables, fruits, legumes and pulses, nuts and wholegrains, encouraging the move to more plant-based ingredients and products in the product portfolio. Positive nutrients include proteins, omega-3 fatty acids, fibers and micronutrients. These nutrients were selected to ensure most common micronutrient deficiencies are tackled, plant-based consumer nutritional needs are met and to promote the benefits of moving towards a more plant-based diet [45]. By 2025, we have committed to double the number of products delivering positive nutrition across the portfolio. Standards for positives and nutrients to limit are separate sets, to avoid the compensation of nutrients to limit by positive ingredients and nutrients. With both sets of standards, we continue to improve the nutrition quality of our portfolio for a positive impact on public health.

## 5. Conclusions

The revised Unilever standards for nutrients to limit were set by translating WHO nutrient guidelines into product group specific standards, considering the role of the product group in the diet, including appropriate serving size and frequency of consumption, and their contribution to the WHO dietary guidelines. The modelling results show that reformulation towards these standards would be impactful, moving nutrient intakes closer to WHO nutrient guidelines in all countries. As these science-based standards can drive substantial reductions in energy, sodium, SAFA and sugar content in products, they may serve as a positive example in discussions on nutrient profiling and standard setting. With these standards, Unilever will continue to improve the nutritional quality of its products, with the aim to help people globally to achieve a healthier diet.

## Figures and Tables

**Table 1:** Daily values used based on WHO guidelines.

Nutrient	Daily Value
Energy	2000 kcal/day
Sodium	2000 mg/day (5 g salt/day)
SAFA	10 en% * = 20 g/day
Free sugars	10 en% * = 50 g/day

* en% = percentage of energy.

**Table 2:** Food consumption surveys and food composition tables used in the modelling.

Country	UK	France	Brazil	US	China
Survey name	UK National Diet and Nutrition Survey (NDNS)	French dietary survey on the general population (INCA3)	Inquérito Nacional de Alimentação (INA)	National Health and Nutrition Examination Survey (NHANES)	China Health and Nutrition Survey (CHNS)
Survey period	2016/2017 and 2018/2019	2015	2017–2018	2017–2018	2004–2011
Age range	≥2 years	2–79 years	≥10 years	≥2 years	≥2 years
Number of participants	4490	3946	46,164	6177	49,464
Dietary assessment method	4× Estimated diet diary	3 × Non-consecutive 24-h records (2–14 years) or 24-h recalls (15–79 years)	2 × 24-h recall	2 × 24-h recall	3× Weighted/measured diet diary
Food composition table used	Composition of foods integrated dataset (CoFID)	ANSES- CIQUAL table	Brazilian Table of Food Composition (TBCA)	Food and Nutrient Database for Dietary Studies (FNDDS)	2004 China Food Composition Tables (CFCT)
Nutrient data available	Energy, sodium, SAFA, added (NMES) and total sugars	Energy, sodium, SAFA and total sugars	Energy, sodium, SAFA and added sugars	Energy, sodium, SAFA, added and total sugars	Energy and sodium

## Data Availability

Restrictions apply to the availability of these data. The food consumption survey data used in the modelling analysis have been described in the article and may be available upon request from the data providers under certain conditions of use.

## Supplementary Materials

The following supporting information can be downloaded at: https://www.mdpi.com/article/10.3390/nu14204289/s1, Table S1: Assumed allocation of energy intake over the day for men (2500 kcal/day), women (2000 kcal/day) and children aged 6–12 years (averaged to 1855 kcal/day), Table S2: Unilever standards for nutrients to limit, Table S3: Standards used for products groups not present in the Unilever portfolio, Table S4: Top contributors to changes in energy intake, Table S5: Top contributors to changes in sodium intake, Table S6: Top contributors to changes in SAFA intake, Table S7: Top contributors to changes in added sugar intake, Table S8: Top contributors to changes in total sugar intake.
