# Supplementary material for: Improving the Nutrient Quality of Foods and Beverages Using Product Specific Standards for Nutrients to Limit Will Substantially Reduce Mean Population Intakes of Energy, Sodium, Saturated Fat and Sugars towards WHO Guidelines
Source: Nutrients. 2022 Oct 14;14(20):4289. doi: 10.3390/nu14204289 (PMC9611440; doi:10.3390/nu14204289)
Supplement: Supplementary file 1 [file nutrients-14-04289-s001.zip › nutrients-1945290-supplementary.pdf]

Supplementary material

Supplementary Table S1: Assumed allocation of energy intake over the day for men (2500 kcal/day), women (2000 kcal/day) and children aged 6-12 years (averaged to 1855kcal/day)

	**Recommended**	**Men**	**Women**	**Children 6-12 years**
Beverages	10%	250 kcal	200 kcal	185 kcal
	The remaining 90% of calories distributed as follows			
Breakfast	20%	450 kcal	360 kcal	330 kcal
Lunch	30%	675 kcal	540 kcal	500 kcal
Dinner	30%	675 kcal	540 kcal	500 kcal
Snacks	20%	450 kcal	360 kcal	330 kcal

Supplementary Table S2 Unilever standards for nutrients to limit

Product group	As sold/as prepared	Energy	Sodium	SAFA	Added Sugars
Main Meals	As prepared	600 kcal/serving	250 mg/100g	1.5 g/100g	1.25 g/100g
Small Meals	As prepared	400 kcal/serving	250 mg/100g	1.5 g/100g	1.25 g/100g
Cereals	As sold	NA	375 mg/100g	2 g/100g	20g/100g
Bread Products	As sold	NA	375 mg/100g	1 g/100g	4g/100g
Spreads - Sweet & Savoury	As sold	NA	720 mg/100g	10 g/100g	17g/100g
Carbohydrate-based Dishes	As prepared	300 kcal/serving	250 mg/100g	1.5 g/100g	1 g/100g
Animal Protein	As sold	300 kcal/serving	675 mg/100g	5 g/100g	1 g/100g
Plant Protein	As sold/as prepared*	300 kcal/serving	450 mg/100g	3 g/100g	2.5 g/100g
Soups	As prepared	NA	265 mg/100g	1.5 g/100g	1 g/100g
Meal Sauces	As sold/as prepared*	NA	340 mg/100g	2 g/100g	2.5 g/100g
Pickled & Fermented Vegetables	As sold	NA	1000 mg/100g	NA	8 g/100g
Water-based Sauces	As sold/as prepared*	NA	750 mg/100g	NA	17 g/100g
Emulsion-based Sauces & Cooking Fats	As sold/as prepared*	NA	750 mg/100g	18% of fat	7 g/100g
Mustards	As sold	NA	2000 mg/100g	18% of fat	7 g/100g
Dish Specific Bouillons & Seasonings	As prepared	NA	265 mg/100g	NA	1 g/100g
Liquid & Paste Bouillons & Seasonings	As sold	NA	4000 mg/100g	NA	40 g/100g
Universal Bouillons & Seasonings	As sold	NA	24,000 mg/100g	NA	20 g/100g
Toppings - Sweet & Savoury	As sold	NA	1250 mg/100g	12.5 g/100g	17 g/100g
Cooking & Baking Agents	As sold	NA	1250 mg/100g	12.5 g/100g	31 g/100g
**Product group**	**As sold/as prepared**	**Energy**	**Sodium**	**SAFA**	**Total Sugars**
Ice-cream & Desserts	As sold/as prepared*	150 kcal/serving	NA	5 g/serving	12 g/serving
Snacks - Sweet & Savoury	As sold/as prepared*	150 kcal/serving	300 mg/serving	3 g/serving	12 g/serving
Kids Ice-cream & Desserts	As sold/as prepared*	110 kcal/serving	NA	3 g/serving	12 g/serving
Kids Snacks - Sweet & Savoury	As sold/as prepared*	110 kcal/serving	300 mg/serving	3 g/serving	12 g/serving
RTD & Concentrated Beverages	As sold/as prepared*	NA	NA	1 g/100g	5 g/100g
Leaf Teas & Herbal Infusions	As prepared	NA	NA	NA	No added sugar
Fruit Juices	As sold/as prepared*	NA	NA	NA	No added sugar
Cereal & Malt-based Beverages	As prepared	NA	NA	1g/100g	6 g/100g**
All Other Products	As sold/as prepared*	NA	100 mg/100g	1 g/100g	2.5 g/100g

**Depending on the product format*

***Excluding sugars from preparation medium (if milk or milk alternative)*

Supplementary Table S3: Standards used for products groups not present in the Unilever portfolio

Product group	As sold/as prepared	Energy	Sodium	SAFA	Added Sugars
Cheese (products)	As sold	NA	675 mg/100g	15 g/100g	NA
Fat spreads (margarine/butter)	As sold	NA	470 mg/100g	33% of total fat	NA
Dairy (excluding cheese)	As sold	NA	NA	NA	NA
Vegetables (fresh and processed)	As sold	NA	NA	NA	NA
Fruit (fresh and processed)	As sold	NA	NA	NA	NA
Supplements	NA	NA	NA	NA	NA
Alcohol	NA	NA	NA	NA	NA
Water	NA	NA	NA	NA	NA
Baby/Toddler foods	NA	NA	NA	NA	NA

Supplementary Table S4: Top contributors to changes in energy intake

	**Absolute change from baseline in energy intake (kcal) by survey**				
	**UK**	**Brazil**	**France**	**China**	**US**
Main meals	-14	-13	-17		-40
Small meals	-24	-59	-47	-11	-122
Carbohydrate-based Dishes	-7	-9	-9	-314	-12
Animal Protein		-5		-45	-8
Plant Protein				-6	
Ice-cream & Desserts	-5		-7		
Snacks - Sweet & Savoury	-22	-17	-76	-2	-56
**Sum of top 5 contributing product groups**	**-72**	**-103**	**-156**	**-378**	**-238**
**Change from all Product Groups**	**-81**	**-105**	**-162**	**-378**	**-242**
**% contribution from top 5 contributors**	**89%**	**98%**	**96%**	**100%**	**98%**

Supplementary Table S5: Top contributors to changes in sodium intake

	**Absolute change from baseline in sodium change (mg) by survey**				
	**UK**	**Brazil**	**France**	**China**	**US**
Main meals	-20	-40	-75		-139
Small meals	-31	-195	-122	-19	-261
Bread Products	-29	-167	-313	-17	-69
Carbohydrate-based Dishes		-28		-14	-40
Animal Protein	-72	-85	-77	-55	-111
Soups			-30		
Pickled & Fermented Vegetables				-93	
Cheese (products)	-10				
**Sum of top 5 contributing product groups**	**-162**	**-515**	**-617**	**-198**	**-620**
**Change from all Product Groups**	**-242**	**-559**	**-685**	**-208**	**-744**
**% contribution from top 5 contributors**	**67%**	**92%**	**90%**	**95%**	**83%**

Supplementary Table S6: Top contributors to changes in SAFA intake

	Absolute change from baseline in saturated fat change (g) by survey			
	**UK**	**Brazil**	**France**	**US**
Main meals	-0.3	-0.6		-1.4
Small meals	-0.9	-1.3	-1.8	-3.4
Bread Products		-0.2		
Carbohydrate-based Dishes				-0.6
Animal Protein	-0.5	-0.8	-1.3	-0.7
Snacks - Sweet & Savoury		-0.1	-1.1	-0.8
Cheese (products)	-0.7		-0.8	
Fat spreads (margarine/butter)	-1.1		-1.4	
**Sum of top 5 contributing product groups**	**-3.5**	**-3**	**-6.4**	**-6.9**
**Change from all Product Groups**	**-5**	**-3.1**	**-9.8**	**-8.3**
**% contribution from top 5 contributors**	**70%**	**97%**	**65%**	**83%**

Supplementary Table S7: Top contributors to changes in added sugar intake

	Absolute change from baseline in added sugars change (g) by survey		
	**UK**	**US**	**Brazil**
Small meals		-1.2	
Cereals & porridges	-0.8	-0.8	
Bread Products			
Spreads - Sweet & Savoury	-1.5		
Animal Protein		-0.9	
Toppings - Sweet & Savoury		-0.8	
Snacks - Sweet & Savoury	-2	-3.4	-0.3
RTD & Concentrated Beverages	-3	-10.9	
Leaf Teas & Herbal Infusions			
Fruit and Vegetable Juices	-3.6		-1.4
**Sum of top 5 contributing product groups**	**-10.9**	**-18**	**-1.7**
**Change from all Product Groups**	-13.4	-21.2	-1.8
**% contribution from top 5 contributors**	81%	85%	94%

Supplementary Table S8: Top contributors to changes in total sugar intake

	Absolute change from baseline in total sugars change (g) by survey		
	**UK**	**US**	**France**
Small meals		-1.2	
Cereals & porridges	-1.2		
Spreads - Sweet & Savoury	-1.4		-5.2
Animal Protein		-0.9	
Toppings - Sweet & Savoury		-0.9	
Ice-cream & Desserts		-0.9	-1.6
Snacks - Sweet & Savoury	-2.7	-4.1	-4.6
RTD & Concentrated Beverages	-3.1	-11.2	-3.8
Fruit and Vegetable Juices	-3.6		-6.9
**Sum of top 5 contributing product groups**	**-12**	**-19.2**	**-22.1**
**Change from all Product Groups**	**-15.5**	**-22.8**	**-25.6**
**% contribution from top 5 contributors**	**77%**	**84%**	**86%**
